# Biomarkers for cognitive decline in patients with diabetes mellitus: evidence from clinical studies

**DOI:** 10.18632/oncotarget.23284

**Published:** 2017-12-14

**Authors:** Xue Zhao, Qing Han, You Lv, Lin Sun, Xiaokun Gang, Guixia Wang

**Affiliations:** ^1^Department of Endocrinology and Metabolism, The First Hospital of Jilin University, Changchun, 130021, Jilin Province, China; ^2^Hospital of Orthopedics, The Second Hospital of Jilin University, Changchun, 130021, Jilin Province, China

**Keywords:** diabetes, cognitive decline, diagnosis, biomarkers

## Abstract

Diabetes mellitus is considered as an important factor for cognitive decline and dementia in recent years. However, cognitive impairment in diabetic patients is often underestimated and kept undiagnosed, leading to thousands of diabetic patients suffering from worsening memory. Available reviews in this field were limited and not comprehensive enough. Thus, the present review aimed to summarize all available clinical studies on diabetic patients with cognitive decline, and to find valuable biomarkers that might be applied as diagnostic and therapeutic targets of cognitive impairment in diabetes. The biomarkers or risk factors of cognitive decline in diabetic patients could be classified into the following three aspects: serum molecules or relevant complications, functional or metabolic changes by neuroimaging tools, and genetic variants. Specifically, factors related to poor glucose metabolism, insulin resistance, inflammation, comorbid depression, micro-/macrovascular complications, adipokines, neurotrophic molecules and Tau protein presented significant changes in diabetic patients with cognitive decline. Besides, neuroimaging platform could provide more clues on the structural, functional and metabolic changes during the cognitive decline progression of diabetic patients. Genetic factors related to cognitive decline showed inconsistency based on the limited studies. Future studies might apply above biomarkers as diagnostic and treatment targets in a large population, and regulation of these parameters might shed light on a more valuable, sensitive and specific strategy for the diagnosis and treatment of cognitive decline in diabetic patients.

## INTRODUCTION

Diabetes mellitus has become one of the most troubling health problems along with the rapid development of social economy. Data from International Diabetes Federation (IDF) showed that that prevalence of diabetes and impaired glucose tolerance (IGT) might reach 8.0% and 10.1% in 2035. As for old people, over 26% of Americans (> 65 years old) were suffering from diabetes mellitus based on the report from American Diabetes Association (ADA) [1]. Moreover, the increased prevalence of diabetes is closely associated with increased risk of all-cause mortality, including ischemic heart disease, stroke and cancer.

Diabetes is known as an important risk factor for cognitive decline and dementia [2–4]. Comparing with patients without diabetes, diabetic patients had 39% increased risk of Alzheimer’s disease and 47% increased risk of dementia [5, 6]. Multiple changes on brain metabolites and brain structures have been reported in patients with diabetes [7]. Thus, the prevalence of cognitive decline is closely linked to diabetes and its progression. However, the cognitive impairment induced by diabetes is often underestimated and kept undiagnosed, leading to thousands of diabetic people suffering from the worsening memory. Thus, targeting diabetes-related brain dysfunction and to explore valuable biomarkers for cognitive decline at early stage are critical problems to be solved urgently [8].

**Figure 1:**
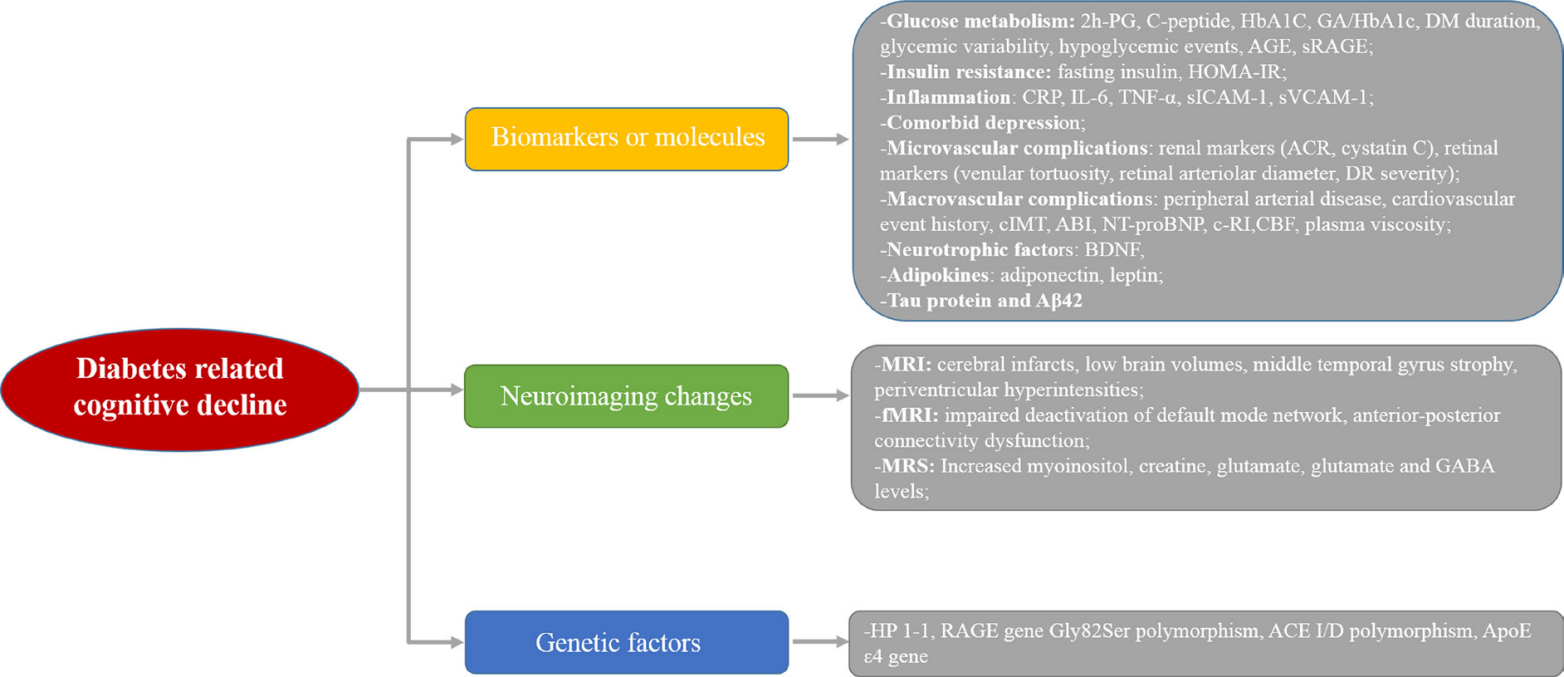
The diagram of possible biomarkers in diabetes-related cognitive decline

Studies have reported that multiple molecules or risk factors were associated with cognitive decline in patients with diabetes. Most reviews just summarized limited relevant biomarkers, few articles comprehensively summed up available evidence on cognitive decline in diabetes. Based on above, we aimed to explore the efficient biomarkers related to early cognitive decline in patients with diabetes and thoroughly summarized available relevant articles, which might be useful for the diagnosis and therapy monitoring of cognitive impairment in patients with diabetes. After carefully analysis, the biomarkers or risk factors of cognitive decline in diabetic patients could be classified into the following three aspects: serum molecules or relevant complications, functional or metabolic changes by neuroimaging tools, and genetic variants (Figure 1). More concretely, parameters related to glucose metabolism, inflammation, comorbid depression and vascular diseases, as well as adipokines have shown significant alterations at the early stage of cognitive decline in diabetes [9–11]. Modern brain imaging technology provides a good platform to detect slight changes in cerebral metabolism and brain structures, such as functional magnetic resonance imaging (fMRI) and magnetic resonance spectroscopy (MRS) [12]. All these early-warning signals presented during cognitive decline process in diabetic patients should be paid great attention [8]. These molecules or changes also shed light on the pathogenesis of diabetes-associated brain damages and the possibility of therapy monitoring in the near future.

### Early biomarkers for cognitive decline in diabetes

#### Association of changed molecules related to glucose metabolism with cognitive decline in diabetic patients

Glucose metabolism is the most frequently affected factor during the development of diabetes. The poor glycemic control in diabetic patients could accelerate the appearance of micro/macro vascular complications. Factors linked to glucose metabolism were reported to be associated with cognitive impairment in diabetic patients (Table 1). Zhou *et al.* [13] explored the clinical characteristics related to cognitive decline on 114 diabetic patients with mild cognitive impairment (MCI) and 83 subjects with normal cognition. Negative correlations were observed between MMSE scores and 2h-glucose level (*P* < 0.001) as well as C-peptide (*P* = 0.001), and these parameters were independent risk factors for impaired cognition in patients with diabetes. Besides these, factors such as older age, female, higher HbA1c level, DM duration and poorer education were correlated with severe cognitive decline in diabetic patients in other studies [14, 15–17]. Study from Kinoshita *et al.* [18] also revealed that glycol-albumin(GA)/HbA1c ratio might be an easy and helpful biomarker for cognitive performance in 88 elderly Japanese diabetic patients. Meanwhile, some studies reported that the fluctuation of glucose level was related to cognition function. Cui *et al.* provided the evidence on the impacts of glycemic variability (GV) on brain atrophy as well as cognitive decline degree in 26 diabetic patients based on 72h continuous glucose monitoring data [19]. The results showed less gray matter and poorer learning and memory function were found in patients with greater glycemic variability (*P* < 0.05). Thus, to keep glycemia in a stale status without great variability is a good way to prevent cognitive decline in diabetes managements.

**Table 1:** Details about the studies focusing on the relationship between glucose parameters and cognitive function in diabetic patients

Study	Population	Design	Number	Mean age	Glucose variables	Cognitive measure	Association with cognition
Zhou et al [13]	T2DM; China	Cross-sectional, observational	197	66.92 ± 8.95	FPG; 2h-PG; FINS; 2h-INS; FCP; HbA1c	MMSE; AVLT; CDR; SCWT	Negative correlation between 2h-PG and FCP and MMSE score.
Moulton et al [14]	T2DM; UK	Cross-sectional, observational	1680	56.10 ± 11	FCP; HbA1c; age; gender; BMI;	TICS-M	Negative association between age, female,HbA1c and TICS-M score
Bruce et al [15]	T2DM; Australia	Retrospective, observational	335	57.50 ± 9.2	FCP; age; DM duration; HbA1c	MMSE; CDR	Age as independent risk factor for cognitive decline; negative correlation between cognition and FCP and DM duration
Kinoshita et al [18]	T2DM; Japan	Cross-sectional, observational	88	74.92 ± 6.44	HbA1c; FPG; FINS; GA;GA/HbA1c	MMSE; HDS-R	GA/HbA1c as independent risk factor for cognitive decline;
Cui et al [19]	T2DM; US	Cross-sectional, observational	69	65.40 ± 9.2	FPG; HbA1c; DM duration; CGM	MMSE; HVLT	Negative correlation between greater glycemic variability and cognition
Ma et al [17]	T2DM; China	Cohort study	1480	75.3 ± 5.9	HbA1c; age; gender; DM duration; DM treatment	Not shown	age > 75 years and longer durations of diabetes as major risk factors for MCI and dementia
Chin et al [22]	T2DM; Korea	KNDP cohort study	4540	67.5 ± 5.5	FPG; HbA1c; hypoglycemic events	Not shown	Linear trend between hypoglycemic events and risk of dementia
Ryan et al [23]	T1DM; US	Cross-sectional, observational	244	55.2 ± 8.3	HbA1c; hypoglycemic events	Mental efficiency tests; nonverbal memory tests; verbal memory tests	Severe hypoglycemic events associated with poorer cognitive function
Wang et al [24]	T2DM; China	Cross-sectional, observational	167	60.15 ± 7.47	sRAGE; AGE-P; HbA1c; FPG; FCP; 2h-CP	MoCA; DST; TMT; CDR; CDT; ST; VFT	Negative correlation for sRAGE; Positive correlation for AGE-P with cognitive function
Ciebiada et al [25]	T2DM; Poland	Cross-sectional, observational	276	73.6 ± 4.8	Serum AGEs, RAGE	MoCA	Increased AGEs, RAGE in MCI patients

Notes: T2DM: type 2 diabetes mellitus; FPG: fasting plasma glucose; 2h-PG: 2h-psprandial glucose; FINS: fasting insulin; FCP: fasting C-peptide; BMI: body mass index; GA: glycoalbumin; sRAGE: soluble receptor for AGEs; AGE-P: Advanced glycation end products-peptides; MMSE: Mini-Mental State Examination; AVLT: Auditory Verbal Learning Test; CDR: Clinical Dementia Rating; SCWT: Stroop Color Words Test; TICS-M: telephone interview for cognitive status; HDS-R: Hasegawa dementia scale-revised; HVLT: Hopkins Verbal Learning Test–Revised; KNDP: Korea National Diabetes Program; TMT: Trail Making Test; DSST: Digit Symbol Substitution Test; MoCA: Montreal cognitive assessment; DST: digit span test; CDT: the clock drawing test; ST: similarities test; VFT: verbal fluency test; MCI: mild cognitive impairment.

Uncontrolled glucose is usually accompanied by frequent hypoglycemic events [20]. Studies have shown that hypoglycemic episodes were closely related to neural damages in the regions of hippocampus and cerebral cortex [21]. Chin *et al.* [22] presented their latest results on the elderly Korean diabetic patients (*n* = 1,957), illustrating that patients with frequently hypoglycemic events had a significantly higher risk of developing dementia (*P* = 0.0286). Besides, the increased dementia risk is related to more severe hypoglycemic events. Thus, there is a vicious circle between dementia and diabetes, contributing to the aggravation of diabetes strikingly as a result. Similar results were reported by Ryan *et al.* [23], they demonstrated negative relationship between cognitive scores and the hypoglycemia episode in the past year (β = –0.360). Thus, to prevent the appearance of hypoglycemic events is an effective way to protect diabetic patients from suffering worsening cognitive function. Future studies should expand the sample size and follow-up duration to validate above findings.

Advanced glycation end products (AGEs) refer to a group of chemicals or molecules formed by the non-enzymatic glycation of proteins, lipids, and nucleic acids. Excessive accumulations of AGEs are closely related to the appearance of vascular complications in diabetes. The receptor for AGE, which is RAGE, can mediate the effects of AGEs contributing to serious damages in different organs during the diabetes progression. However, the role of AGE and RAGE in diabetes related cognitive decline is unclear. Wang *et al.* [24] revealed decreased soluble RAGE (sRAGE) level and increased AGE-peptide level in 82 diabetic patients with MCI (*P* < 0.01). Negative association was found between sRAGE level and trail making test-B (TMT-B, *P* = 0.002). Another study from Gorska-Ciebiada *et al.* [25] showed increased AGE and RAGE levels in MCI patients, which were positively correlated with HbA1c level, but inversely correlated with MoCA scores. Thus, there were increased AGEs and RAGE levels in diabetic patients with cognitive decline, indicating AGEs and RAGE might be applied as biomarkers for the appearance of cognitive decline in diabetes. However, the specificity and sensitivity of these markers were unknown.

### Association between insulin resistance or hyperinsulinemia with cognitive decline in diabetes

Insulin resistance (IR) or hyperinsulinemia have generated increasing attention due to its critical role in the development of cognitive decline or dementia [26]. Clinical studies have shown the close link between IR and cognitive dysfunction in diabetic patients (Table 2). Ma *et al.* [17] explored their research on 212 old patients with T2DM in two subgroups (cognitive impairment *n* = 100; normal cognitive group *n* = 112). Compared with normal cognitive group, patients with impaired cognitive function presented lower education level, higher fasting insulin level and higher HOMA-IR [(6.9 ± 1.7) vs (3.9 ± 0.9), *p* < 0.01]. Thus, IR and fasting could be independent risk factors for cognitive dysfunction. To discover the impact of the severity of IR on cognitive function, Hishikawa *et al.* [27] enrolled 182 outpatients with DM and found significantly decreased MoCA scores in ‘naming’, ‘read list of letters’ and ‘delayed recall’ aspects along with the deteriorating of HOMA-IR (HOMA-IR: < 1.2; 1.2–12; > 12). Similar results were reported by Zhong *et al.* [28]. Yuan and his colleague reported that elderly patients with hyperinsulinemia (FINS > 10.5mU/L; *n* = 180) and IR (HOMA-IR > 2.31; *n* = 192) had lower cognitive test scores in MMSE, MoCA, CDR, orientation, delayed memory, and attention/calculation domains. Other studies also revealed negative association between HOMA-IR and cognitive impairment [29–31]. Neergaard *et al.* [32] discovered that patients with HOMA-IR > 2.6 held 47% larger possibility to suffer from cognitive decline in the Prospective Epidemiological Risk Factor study (*n* = 2,103). Pin Ng *et al.* [33] reported that metabolic syndrome increased the risk of MCI and its progression to dementia in 1519 participants (HR: 1.46). However, inconsistent results were demonstrated by Brutto *et al.* [34] and Geijselaers *et al.* [35], they found there was no association between the existence of metabolic syndrome and cognitive impairment. The explanation for this inconsistency should be?

**Table 2:** Details about the studies focusing on the relationship between insulin resistance and cognitive function in patients with diabetes

Study	Population	Design	Number	Mean age	Insulin resistance	Cognitive measure	Association with cognition
Ma et al. [26]	T2DM; China	Cross-sectional, observational	212	70.7 ± 9.73	FPG, FINS, HOMA-IR, BMI	MMSE	FINS, HOMA-IR and education level as independent risk factors for cognitive decline;
Hishikawa et al. [27]	T2DM; Japan	Cross-sectional, observational	182	64.7 ± 18.0	FPG; HbA1c; HOMA-IR	MMSE; HDS-R; MoCA; FAB;	Negative association between HOMA-IR and MoCA score
Zhong et al. [28]	T2DM; China	Cohort study	328	57.50 ± 9.2	FPG; FINS; HOMA-IR	MMSE, MOCA, CDR, GDS; ADL	Negative correlation between FINS, HOMA-IR and MMSE score as well as delayed memory
Umegaki et al. [29]	DM; Japan	Cross-sectional, observational	444	72.4 ± 4.7	HbA1c; HOMA-IR	MMSE, semantic fluency, digit span, digit symbol, TMT-A, TMT-B	Negative correlation between HOMA-IR and logical memory 2 and MMSE score;
Ekblad et al. [30]	IR; Finland	Cohort study	5935	52.5 ± 14.7	FPG; HbA1c; TG; HOMA-IR; HDL	VFT; WLL; WLDR; RT; VC	Negative association between HOMA-IR and verbal fluency test score in women, not men
Willette et al. [31]	IR; US	Cross-sectional, observational	280	75.23 ± 7.13	FPG; FINS; HOMA-IR; BMI; Age	MMSE	Hypermetabolism in medial temporal lobe in MCI patients; Positive association between FDG level and IR in MTL region.
Geijselaers et al. [35]	IR; Netherlands	Cross-sectional, observational	641	62 ± 8	FINS; C-peptide; HOMA-IR; BMI; Age; FPG; HbA1c	memory, executive function, attention, information processing speed	FINS, C-peptide, HOMA-IR not associated with cognitive performance
Neergaard et al. [32]	MetS; Denmark	Cohort study	2103	65.40 ± 9.2	FPG; HbA1c; BMI; HOMA-IR;FINS; TG; HDL; BP	Short Blessed Test; category fluency test	Negative correlation between FPG, HOMA-IR and cognitive function
Brutto et al. [34]	MetS; US	Cross-sectional, observational	212	69.2 ± 7.2	FPG; BP; TG; age; BMI; education;	MoCA;	No correlation
Ng et al. [33]	MetS; Singapore	Cohort study	1519	64.9 ± 6.8	BP; lipid profiles; age; education;	MMSE; memory; language; attention;	Increased MCI risk in patients with MetS

Notes: T2DM: type 2 diabetes mellitus; MetS: metabolic syndrome; FPG: fasting plasma glucose; 2h-PG: 2h-psprandial glucose; FINS: fasting insulin; BMI: body mass index; TG: triglyceride; BP: blood pressure; HOMA-IR; MMSE: Mini-Mental State Examination; CDR: Clinical Dementia Rating; HDS-R: Hasegawa dementia scale-revised; TMT: Trail Making Test; MoCA: Montreal cognitive assessment; FAB: frontal assessment battery; VFT: verbal fluency test; MCI: mild cognitive impairment; WLL: word-list learning test; WLDR: word-list delayed-recall test; RT: reaction-time test; VC: visual choice reaction-time test.

In conclusion, insulin resistance or hyperinsulinemia played an important role in the cognitive decline of diabetic patients. Diabetes-induced cognitive impairment might occur at the early stage of diabetes, even in IR patients without diabetes. Preventing strategies should be performed in IR patients as soon as possible. As for the mechanism of IR induced neuropathy, available studies revealed that IR might affect hippocampus plasticity, APP metabolism, tau protein metabolism and inflammation reaction [26, 36]. All these factors might synergistically contribute to the deteriorated cognitive function in diabetic patients. However, the interactions between these influencing factors remain to be illustrated in the near future.

### Association between inflammatory factors and cognitive decline in diabetes

Chronic inflammation is considered to play an important role in the development of cognitive impairment. Studies have shown the existence of enhanced inflammation in the brain of patients with dementia [37]. In patients with diabetes, cognitive decline is associated with remarkable changes in inflammatory molecules (Table 3). Marioni *et al.* explored the circulating inflammation markers (CRP, IL-6 and TNF-α) in 1,066 patients with diabetes, demonstrating that IL-6 and TNF-α were associated with cognitive function. Moreover, higher level of inflammation markers were related to poorer cognitive scores after sex- and age- adjustment (*P* < 0.05) [38]. Gorska-Ciebiada *et al.* [39, 25] revealed increased CRP, IL-6 and TNF-α in patients with both diabetes and MCI. The inflammation markers were positively correlated with HbA1c and negatively correlated with MoCA scores. Notably, diabetic patients with both MCI and depression presented highest levels of inflammation markers (CRP, IL-6 and TNF-α). As for other inflammation factors, IL-1β was increased in patients with MCI group when compared with normal controls [40]. Besides, soluble intercellular adhesion molecule 1 (sICAM-1), soluble vascular adhesion molecule 1(sVCAM-1) and hs-CRP in diabetic patients with MCI and diabetic patients with both MCI and depression [41]. And diabetic patients with both MCI and depression presented highest sICAM-1 and sVCAM-1 levels when compared to diabetic patients with depression, indicating inflammation factors plays an important role in the development of MCI in diabetic patients with depression. Besides direct influences on cognition function, inflammation can also affect cerebral vasoregulation. In 65 diabetic patients, Chung *et al.* [42] found that increased soluble intercellular, vascular adhesion molecules and hs-CRP were correlated with decreased cerebral vasoreactivity and vasodilation (*P* = 0.007–0.048), illustrating that inflammation could influence the brain vasoregulation and accelerate the progression of cognitive impairment in diabetes. To further validate the role of inflammation in cognitive impairment, Lavielle *et al.* [43] performed their study on 1,712 patients with new diagnosis of T2DM, showing that rheumatoid arthritis and asthma, two chronic inflammatory diseases, were risk factors for cognitive impairments in diabetic patients. Overall, inflammation is an important marker for diabetes-induced cognitive decline. However, details about variation range of these inflammatory factors to diagnose cognitive impairment are required to be explored in the near future. Moreover, since inflammation exists widely in chronic decreases, the predictive power of inflammatory markers, referring to the sensitivity and specificity, in the diagnosis and therapy monitoring for cognitive decline in diabetes deserve more concerns.

**Table 3:** Details about the studies focusing on the relationship between inflammation factors and cognitive function in diabetic patients

Study	Population	Design	Number	Mean age	Inflammation factors	Cognitive measure	Association with cognition
Marioni et al [38]	T2DM; UK	Cross-sectional, observational	1066	67.9 ± 4.2	CRP; TNF-α; IL-6	MR; LNS; VFT; DST; TMT; LM; FACES	Higher TNF-α and IL-6 associated with poorer cognitive function.
Gorska-Ciebiada et al [39]	T2DM; Poland	Cross-sectional, observational	276	73.6 ± 4.8	CRP, IL-6, TNF-α	MoCA	Negative association between CRP, TNF-α and MoCA score
Gorska-Ciebiada et al [40]	T2DM; Poland	Cross-sectional, observational	194	73.2 ± 4.5	IL-1β	MoCA	Increased IL-1β correlated with increased risk of MCI
Gorska-Ciebiada et al [41]	T2DM; Poland	Cross-sectional, observational	219	74.92 ± 6.44	hs-CRP; sICAM-1; SVCAM-1;	MoCA	Increased sICAM-1 and sVCAM-1 correlated with increased risk of MCI
Chung et al [42]	T2DM; US	Cross-sectional, observational	69	65.40 ± 9.2	hs-CRP; sICAM-1; SVCAM-1;	MMSE; HVLT-R: IADL	Negative correlation between hs-CRP; sICAM-1; SVCAM-1 and cerebral vasoreactivity, vasodilation and cognition
Lavielle et al [43]	T2DM; Mexico	Cross-sectional, observational	1712	51 ± 11	Rheumatoid arthritis; asthma	CDT; Verbal fluency; Calculation	Rheumatoid arthritis and asthma as risk factors for MCI

Notes: DST: digit symbol test; FACES: Faces and Family Pictures Subtest; HADS: Hospital Anxiety and Depression Scale; LM: logical memory; LNS: letter-number sequencing; MR: matrix reasoning; TMT: Trail Making Test-Part B; VFT: Verbal Fluency Test; sICAM-1: soluble intercellular adhesion molecule 1; sVCAM-1: soluble vascular adhesion molecule 1; HVLT-R: Hopkins Verbal Learning Test–Revised; IADL: Instrumental Activities of Daily Living; MMSE: Mini-Mental State Examination; CDT: The clock drawing test free-hand format; MCI: mild cognitive impairment.

### Comorbid depression as an important risk factor for cognitive decline in diabetes

Both depression and diabetes are known as important risk factors for cognitive impairment in elderly people [44]. Decreased hippocampal volume was observed in depressed patients when compared with non-depressed patients [45]. However, if the presentence of depression increased the risk of cognitive decline in diabetic patients is unclear. Here, we summarized available studies on the relationship between depression and cognitive impairment in diabetic patients (Table 4). Watari *et al.* explored the cognitive performance in diabetic patients with depression, revealing that depression negatively correlated with cognitive function [46]. Patients with depression presented worse performance in attention/information processing speed and executive functioning (*P* < 0.05). Similar results were presented by Koekkoek and colleagues, demonstrating that the occurrence of depression increased two-fold risk of cognitive decline in patients with diabetes [47]. Johnson *et al.* [48] and Downer *et al.* [49] both detected the impact of depression on diabetes-related cognitive decline in Mexican Americans in large populations. They revealed that comorbid depression in diabetic patients significantly increased the risk of MCI and dementia. A prospective cohort study of 3837 patients explored the influence of comorbid depression on risk of dementia in diabetic patients [50]. After 5-year follow up, the incidence of MCI in diabetic patients was 21.5 per 1,000 person-years, comparing with 11.8 per 1,000 person-years in patients with diabetes alone (*P* < 0.05). These results showed a significant increased risk of dementia in depressed diabetics. Two years later, Katon *et al.* reported their updated data in a larger population (*n* = 19,239), showing a 100% increased risk of dementia in patients with both diabetes and depression comparing with patients with diabetes [51]. Similar results were also found in diabetic patients after stroke [52], the presence of preoperative depression was an important risk factor for postoperative short-term and long-term cognitive impairment in patients undergoing coronary artery bypass graft surgery [53]. However, the mechanism of depression in diabetic patients is controversial. Studies have shown close relationship between mood disturbances and altered subgenual cingulate cortex (SGC) resting-state functional connectivity. van Duinkerken and his colleagues observed a close correlation between decreased SGC to inferior frontal gyrus and frontal pole connectivity and increased depression symptoms [54]. Thus, comorbid depression was an important risk factor for the development of cognitive impairment in patients with diabetes, suggesting the possibility of beneficial effects of antidepressants treatment on the normalization of cognitive function. To confirm this suspect, Guo *et al.* performed 6-month intervention of metformin in diabetic patients and found significant improvements in depression symptoms, which was associated with enhanced cognitive function [55]. However, the underlying mechanism of metformin in improving depression and cognition was unestablished. It is also unclear if other hypoglycemic drugs have similar beneficial effects on depression and cognitive impairment in diabetic patients. Future studies should focus on the monitoring of early diagnosis on depression in diabetic patients and explore effective antidepressants treatments to protect diabetic patients from mood disorders.

**Table 4:** Details about the studies focusing on the relationship between depression and cognitive function in diabetic patients

Study	Population	Design	Number	Mean age	Depression measure	Cognitive measure	Association with cognition
Johnson et al [48]	T2DM; US	Cross-sectional, observational	2436	64.4 ± 10.6	GDS-30	MMSE; WAIS-III;TMT;WMS-III	Comorbid depression and age as risk factors for cognitive impairment
Swardfager et al [52]	T2DM; Canada	Cohort study,	342	67 ± 13.5	CES-D	MoCA;	Diabetes and depressive symptoms increases risk of severe cognitive impairment
Downer et al [49]	T2DM; US	Retrospective cohort study	2756	73.2 ± 6.1	CES-D	MMSE; ADL	Depression correlated with greater cognitive decline
van Duinkerken et al [54]	T1DM; Netherlands	Cross-sectional, observational	153	40 ± 9.3	CES-D	Memory; information processing speed; executive function; attention; motor speed; psychomotor speed	Increased depressive symptoms was related to poorer general cognitive ability and lower subgenual cingulate cortex functional connectivity
Koekkoek et al [47]	T2DM; Netherlands	Cross-sectional, observational	225	76.8 ± 5.0	CES-D	TYM; SAGE	Depression occurred twice as often in patients with cognitive impairment
Kadoi et al et al [53]	T2DM; Japan	Cross-sectional, observational	90	65 ± 9	21-item Beck depression inventory	MMSE; TMT-A;TMT-B; digit span forward; grooved pegboard	Preoperative depression as a risk factor for postoperative short-term and long-term cognitive dysfunction
Watari et al [46]	T2DM; US	Cross-sectional, observational	74	57.9 ± 11.1	HAM-D	MMSE; Attention and information processing speed; TMT-A; WAIS-III	Depression negatively impacts cognitive performance
Guo et al [55]	T2DM; China	Prospective study	58	54.7 ± 7.3	MADRS; HRSD-17	WMS-R; DSM-IV	Metformin treatment improved cognitive function and has antidepressant behavioural effects
Katon et al (a) [50]	T2DM; US	Prospective cohort study	3837	63.2 ± 13.2	PHQ-9	ICD-9	Patients with major depression with an increased risk of development of dementia (7.9% vs 4.8%)
Katon et al. (b) [51]	T2DM; US	Prospective cohort study	19239	58.8 ± 10.0	PHQ-8	ICD-9	Diabetic patients with depression had a 100% increased risk of dementia

Note: MMSE: Mini-Mental State Examination; TMT: Trail Making Test; MoCA: Montreal cognitive assessment; CES-D: Center for Epidemiological Studies Depression; GDS-30: Geriatric Depression Scale (GDS-30); TYM: Test Your Memory; Self-Administered Gerocognitive Examination; HAM-D: Hamilton Rating Scale for Depression; WMS-R, Wechsler Memory Scale–Revised; MADRS, Montgomery Asberg Depression Rating Scale; MADRS: Montgomery–Asberg Depression Rating Scale; HRSD-1:17-item Hamilton Rating Scale for Depression; DSM-IV: Diagnostic and Statistical Manual of Mental Disorders–Fourth Edition. PHQ-9: Patient Health Questionnaire

### Association between micro-/macrovascular complications related markers and cognitive decline

#### Microvascular complications related markers

#### Renal markers

Diabetes could induce various complications in different organs, especially in kidney, which is called diabetic kidney disease (DKD). Renal function impairment is shown to be related to cognitive decline (Table 5), which increases the mortality and impairs the life quality of patients significantly [56, 57]. Murray *et al.* [58] explored the correlation between three renal function markers and cognition function, showing that higher level of albumin/creatinine ratio (ACR) was related to greater decreases in DSS test scores (*P* = 0.001) and RAVLT scores (*P* = 0.006). Similar trend was found between cystatin C and cognitive tests. However, no significant correlation was observed between estimated GFR and ant cognitive tests (*P* > 0.05). Another study from Zhang *et al.* [59] revealed significant increases of ACR and cystatin C in diabetic patients with MCI, compared with diabetic patients without MCI. Positive association between increased cystatin C and the risk of cognitive impairment was found in diabetic patients. Moreover, the add on of cystatin C to traditional risk factors provided a relative high diagnostic value with area under the curve (AUC) as 0.91, suggesting the possibility of cystatin C as biomarker for cognitive decline in diabetes. In the study by Kawamura [60], correlation between ACR and word recall scores was reported. When estimated GFR decreased, patients presented decreased MMSE and DSS scores. Thus, monitoring renal markers regularly in patients with diabetes not only provide the information about renal function or kidney disease progression, but also shed light on the effective prevention of cognitive decline.

**Table 5:** Details about the studies focusing on the relationship between micro-/macro-vascular disease markers and cognitive function in diabetic patients

Study	Population	Design	Number	Mean age	Vascular markers	Cognitive measure	Association with cognition
Murray et al [58]	T2DM; UK	Cross-sectional, observational	2968	62.47 ± 5.7	eGFR; ACR; cystatin C	MMSE; RAVLT; DSST; Stroop Test	Higher ACR and cystatin C associated with poor cognitive function
Zhang et al [59]	T2DM; China	Cross-sectional, observational	357	66.58 ± 9.8	eGFR; ACR; cystatin C	MoCA	Elevated cystatin C associated with increased risk of MCI
Kawamura et al [50]	T2DM; Japan	Cohort, observational	67	74.60 ± 5.5	eGFR; ACR;	MMSE; DSST; Stroop Test	Decreased eGFR correlated with poor cognitive function
Naidu et al [61]	T2DM; UK	Case-control, observational	137	50–70	retinal vessel calibre, arterio-venous ratio, retinal fractal dimension, retinal vessel tortuosity	TICSM	Positive correlation between higher venular tortuosity and cognitive decline
Ryan et al [23]	T1DM; US	Cross-sectional, observational	244	55.2 ± 8.3	retinal vessel diameters (CRAE, CRVE)	Mental efficiency tests; nonverbal memory tests; verbal memory tests	Negative correlation for CRVE; Positive correlation for CRAE with cognitive function
Nwaobi et al [62]	T2DM; UK	Cross-sectional, observational	380	64.8 ± 10.8	DR severity	ACE-R; MMSE; Mini-Cog scores	Negative relationship between severity of DR and cognition function
Moreira et al et al [64]	T2DM; Brazil	Cross-sectional, observational	149	65.73	HbA1c; hypoglycemic events	NDS; NSS	No association
Bruce et al [67]	T2DM; Australia	Cross-sectional, observational	302	75.70 ± 4.6	peripheral arterial disease	MMSE; IQCODE	Peripheral arterial disease as an independent risk factor for dementia
Feinkohl et al [68]	T2DM; UK	Cohort study	832	67.69 ± 4.1	CV event; cIMT; ABI; NT-proBNP	MMSE; BVFT; LM; DSC; MR; MHVS	Positive correlation between stroke history, ABI, cIMT, NT-proBNP and accelerated cognitive impairment
Chen et al [69]	T2DM; China	Cross-sectional	157	55 ± 7	HbA1c; age; gender; lipid profiles; cIMT; c-RI; C-peptide	MoCA	c-RI, C-peptide, hypertension history as determinants for MoCA scores
Jansen et al [70]	T2DM; Netherlands	Cross-sectional, observational	80	61.85 ± 8	cerebral blood flow	MMSE; Cumulative cognition score	No association
Marioni et al [71]	T2DM; UK	Cross-sectional, observational	1066	67.9 ± 4.2	plasma viscosity; haematocrit	FACES; DST; LNS; LM;VFT; TMT; MHVS	Negative correlation for plasma viscosity; Positive correlation for haematocrit with cognitive function
Mehrabian et al [72]	T2DM; Bulgaria	Cross-sectional, observational	59	56 ± 6.8	carotid-femoral pulse wave velocity	MMSE; FCSRT; BNT; DSS; TMT;	Positive association between CF-PWV and cognitive function

Notes: eGFR: estimated glomerular filtration rate; ACR: albumin/creatinine ratio; MMSE: Mini Mental State Examination; RAVLT: Rey Auditory Verbal Learning Test; DSST: Digit Symbol Substitution Test; TICSM: Telephone Interview for Cognitive Status; CRAE: central retinal arteriolar equivalent; CRVE: central retinal venular equivalent; ACE-R: Addenbrooke’s Cognitive Examination-Revised; NDS: Neuropathy Disability Score; NSS: Neuropathy Symptom Score; MCI: mild cognitive impairment; IQCODE: Informant Questionnaire for Cognitive Decline in the Elderly; CV: cardiovascular event history; Cimt: carotid intima-media thickness; ABI: ankle brachial index; NT-proBNP: serum N-terminal probrain natriuretic peptide; BVFT: Borkowski Verbal Fluency Test; LM: The Logical Memory; DSC: The Digit Symbol Coding; MR: Matrix Reasoning; MHVS: Mill Hill Vocabulary Scale; FCSRT: Free and Cued Selective Reminding Test; BNT: Boston Naming Test.

#### Retinal marker

Studies have shown close relationship between changes in retinopathy and cognitive impairment (Table 5). Naidu *et al.* [61] performed their research on 69 newly diagnosed diabetic patients and 69 healthy control, showing positive relationship between venular tortuosity level and cognitive decline (*P* = 0.013). To investigate the changes in vessel diameters, Ryan *et al.* [23] reported the positive association between retinal arteriolar diameter and metal efficiency. The negative association between retinal venular diameter and metal efficiency was also observed (*P* < 0.05). To explore whether the severity of diabetic retinopathy (DR) is correlated with cognitive impairment, Nwaobi *et al.* [62] divided 380 diabetic patients into non-DR group (*n* = 252) and proliferative diabetic retinopathy group (PDR, *n* = 128), showing that the severity of DR was negatively correlated with cognition function. Interestingly, these results indicated that patients without DR or mild DR presented worse cognitive performance, while patients with PDR exhibited better cognition. Similar results were also reported in animal studies [63]. However, the underlying mechanism for the negative association between DR and cognitive decline was unclear. Since the limited available evidence on DR and cognitive decline, whether the severity of DR can influence the cognitive function remains uncertain. Future studies are required to explore above findings in a larger sample size and longer follow up periods.

#### Peripheral neuropathy marker

Few studies have detected the association between peripheral neuropathy and cognitive performance (Table 5). Moreira *et al.* [64] reported that there was no significant difference in cognitive test scores, such as MMSE, Trail Making Test A and B, between patients with diabetes and patients diabetic peripheral neuropathy (DPN) (*P* > 0.05). No relationship between DPN assessment scores and cognitive scores was found, indicating no obvious correlation between cognitive impairment and DPN. However, due to the small sample size of available few studies, it is hard to get a confirmed conclusion.

### Macrovascular complication related markers

Besides the close relationship between microvascular diseases and cognitive decline in diabetes, obvious influence of larger vessel changes on cognition function were reported by various studies [65, 66] (Table 5). Bruce *et al.* [67] explored the predictors of cognitive decline in 302 elder diabetic patients, showing that peripheral arterial disease is an independent risk factor for dementia (OR:5.35, *P* < 0.05). Feinkohl *et al.* [68] investigated the association between macrovascular disease markers and cognitive decline in 832 diabetic patients by detecting clinical parameters, including cardiovascular event history, carotid intima-media thickness (cIMT), ankle brachial index (ABI), and serum N-terminal probrain natriuretic peptide (NT-proBNP). After four follow-up, the stroke history, ABI, cIMT, NT-proBNP were found to be correlated with accelerated cognitive impairment, indicating the close relationship among atherosclerosis and cardiac markers and cognitive decline. Study by Chen and his colleague presented carotid resistant index (c-RI) was also important determinants for cognitive decline in diabetic patients [69]. Jansen *et al.* [70] revealed that low cerebral blood flow (CBF) in cerebral cortex was related to depression in diabetic patients, but not related to cognitive function. Many reasons can contribute to low CBF in diabetic patients, and factors related to blood rheology might play an important role. To see the impact of blood rheology on cognitive decline, Marioni *et al.* [71] detected the plasma viscosity and haematocrit in 1,066 patients with diabetes. An inverse association between plasma viscosity and cognitive test scores was observed, while a positive association was observed between haematocrit and cognitive test scores (*P* < 0.05). Mehrabian *et al.* [72] performed their research on arterial stiffness and cognitive function, revealing that carotid-femoral pulse wave velocity (CF-PWV) was significantly higher in patients with diabetes compared with controls (*P* < 0.001).

Thus, it is valuable to detect both macrovascular and microvascular diseases in diabetic patients. When diabetic patients present macro/microvascular diseases, much more attention should be paid on the evaluation of cognitive function. Especially for diabetic patients with stroke or cardiac event, the prevention and management of cognitive decline shouldn’t be ignored.

### Association between neurotrophic factors and cognitive decline in diabetes

Neurotrophic factors can regulate memory-related neuroplasticity and modulate neural cell survival, growth and proliferation [73, 74]. Brain-derived neurotrophic factor (BDNF), as one of the most important neurotrophic factors, is mainly expressed in brain tissue, while it can also be detected in the blood. Zhen *et al.* [75] explored the association between BDNF and cognitive impairment in 208 diabetic patients and 212 healthy controls. Diabetic patients presented decreased BDNF level, and decreased BDNF level was correlated with poor cognitive function (*P* < 0.001). Similar results were reported by Ortíz *et al.* [76] in 40 diabetic patients. Thus, low BDNF might be an effective biomarker for diabetes related cognitive impairment.

### Association between adipokines and cognitive decline in diabetes

The incidence of obesity has increased dramatically in the past decades. Studies have shown its intact association with brain dysfunction, cognitive decline and dementia [77]. Adipokines, as hormones secreted from adipose tissue, are considered to be important mediators for obesity-related cognitive impairment [78]. Study by Casares *et al.* [79] investigated that lower adiponectin was related to impaired glucose metabolism and decreased gray matter volume in diabetic patients (*P* < 0.001). Consistent results were demonstrated by Ciebiada *et al.* [40], who revealed decreased adiponectin level in MCI patients. Beyond that, Ciebiada *et al.* found increased leptin level in MCI group, which is another adipokine released by adipose tissue. Labad *et al.* [80] illustrated an inverse association between leptin level and cognitive performance in 1,057 diabetic patients, indicating that higher fasting leptin correlated with poorer cognitive test scores (*P* < 0.02). Thus, adiopkines can be valuable markers for the cognitive impairment, especially for adiponectin and leptin levels. However, the specificity and sensitivity of these markers were unknown, when compared with traditional markers.

### Association between Tau protein and Aβ_42_ and cognitive decline in diabetes

Close relationship between diabetes and Alzheimer’s disease (AD) has been reported in recent years [3, 81]. β-amyloid (Aβ) deposition and Tau hyperphosphorylation are known as classical pathogenic factors of AD [82, 83]. Ouwens *et al.* detected AD-related markers in cerebrospinal fluid (CSF) on 37 patients with T1DM and 15 healthy controls. Higher level of pTau and Aβ42 in CSF were found in T1DM patients (*P* < 0.05). Moreover, increased Tau protein was found to be correlated with declined white matter in right inferior fronto-occipital tract (*P* = 0.002). In study by Moran *et al.* [84], patients with T2DM showed significantly higher total Tau and pTau levels in CSF (*P* = 0.04, *P* = 0.02, respectively). Thus, diabetes can induce the classical pathogenesis of AD, which is reflected by increased Aβ42, Tau and pTau levels. These markers could provide remarkable clues in the development of diabetes-associated cognitive impairment and AD, which can also be used for the assessment of cognitive function in diabetes.

### Neuroimaging as efficient tools to detect cognitive decline in diabetes

#### MRI alterations of cognitive decline in diabetes

Lower brain volume and brain atrophy are considered as features of many neurodegenerative diseases. Studies have shown diabetes-associated cognitive decline could induce structural lesions, which can be reflected by neuroimaging tools [85–87]. Thus, exploring altered structural pattern in diabetic patients with cognitive decline might provide possible mechanisms of neurodegenerative process in diabetes. However, limited studies investigated the brain changes in diabetic patients with cognitive impairment. Moran *et al.* [88] found positive associations between diabetes and cerebral infarcts, lower gray as well as white matter volumes (*P* < 0.05). Female were more susceptive to cerebral complications, when compared with male [89]. Hayashi *et al.* revealed that cognitive impairment was associated with hippocampal atrophy, but not the whole brain atrophy in 61 Japanese diabetic patients [90]. Zhang *et al.* [91] investigated the brain changes in diabetic patients with and without MCI. Positive relationship between atrophy of middle temporal gyrus and increased risk of MCI was observed. Besides gray matter volume, Manschot *et al.* found impaired cognitive function correlated with white matter lesions, cortical atrophy and infarcts (*P* < 0.05) [92]. Moreover, periventricular hyperintensities was reported to be associated with motor speed [93]. Thus, MRI can reflect brain structural changes in patients with diabetes and cognitive decline, although specific affected regions caused by the diabetes were not consistent.

### fMRI alterations of cognitive decline in diabetes

Functional MRI (fMRI) provides a good platform to explore the alterations of brain function in patients with cognitive decline *in vivo* [94, 95]. Studies have shown that functional changes might exist before the appearance of brain structure alterations. Thus, fMRI could be a valuable tool to detect early markers of diabetes-related cognitive decline. Default mode network (DMN) is a region in charge of cognitive tasks. Marder *et al* [96] showed impaired deactivation DMN regions during recognition task in diabetic patients, which was correlated with peripheral plasma glucose level. Chen’s study presented similar results in the DMN regions (including right precuneus and the middle temporal gyrus) during memory tasks [97]. They concluded that the anterior-posterior connectivity dysfunction in diabetes might contributed to cognitive impairment. In diabetic patients with MCI, Yang *et al.* [98] found disturbed intranetwork and internetwork functional connectivity DMN region, CON region, right insula and posterior cerebellum. These dysfunctions were positively correlated with HbA1c and DM duration, indicating the key role of hyperglycemia in the process of cognitive decline. Zeng *et al.* demonstrated consistent results in 28 diabetic patients and found dysfunctional network was related to poorer MoCA scores [99]. Thus, the network organization and functional connectivity in some brain regions could be affected in patients with diabetes, which can be used for the evaluation and diagnosis of cognitive decline.

### MRS alterations of cognitive decline in diabetes

Brain, as one of the target organ affected by diabetes, has shown metabolic dysfunction in various studies [100, 101]. To explore the altered brain metabolites is of great value in detecting cognitive decline at early stage. Magnetic resonance spectroscopy (MRS), as a neuroimaging method, can detect the alterations of neurochemicals related to metabolism and energetics *in vivo* [102]. Wang *et al.* [103] performed their study in 188 T2DM patients and observed altered brain metabolites in hippocampus rather than frontal lobe. Increased myoinositol and creatine levels in hippocampus were correlated with poor cognitive test scores. In Lyoo’s study [104], 123 T1DM patients presented significantly higher glutamate, glutamate and GABA levels in prefrontal area when compared with healthy controls. An inverse association was found between these metabolites and cognitive function. However, inconsistent results were reported by Tiehuis *et al.* [105], they showed there was no relationship between cognitive function and brain metabolites in white matter of patients with diabetes. Based on limited evidence, we could believe there were metabolic dysfunctions in diabetic brain, and these changes were closely related to the appearance of cognitive decline in diabetes. However, the valuable and specific metabolites as biomarkers for cognitive decline in diabetes were not found.

### Genetic factors related to cognitive decline in diabetes

Findings associated with genetic factors for cognitive decline is of great interest for clinicians and researchers. However, available evidence presented controversial results (Table 6). Haemoglobinbinding protein can protect cells from oxidative damage, which is produced from haptoglobin (Hp) gene [106]. Studies have shown that Hp gene presented obvious downregulation in MCI patients when compared with controls [107]. Berroa *et al.* revealed that HP 1-1 carriers showed a stronger relationship between HbA1c and impaired cognitive performance (*P* < 0.01) [108]. These indicated HP 1-1 carriers hold a higher risk for cognitive decline. RAGE gene is one of the important genes in diabetic complications, Gly82Ser polymorphism of RAGE gene was reported to play an important role in AD [109]. Wang *et al.* [24] found RAGE Gly82Ser carriers with lower RAGE concentration than non-carriers in MCI patients (*P* = 0.003). But no significant association was reported between cognitive test scores and 82Gly/Ser genotype (*P* > 0.05). Angiotensin-converting enzyme (ACE) is one of the key genes in RAS system [110]. Studies have shown that Ang II was related to AD in elder patients. Tian *et al.* [111] analyzed different I/D single-nucleotide polymorphisms of ACE gene in T2DM patients (*n* = 210). Patients with MCI presented higher ACE level and ACE activity. However, these was no change in the genotype of ACE I/D polymorphism in these patients. The APOE gene is known to play a key role in lipid metabolism in brain tissue, which is closely related to cognitive function [112]. Xu *et al.* [113] explored the biomarkers for the diagnosis of MCI in diabetic patients (*n* = 694). In their study, older age, ApoE ε4 allele, higher olfactory score and higher rGSK-3β (ratio of total GSK-3β to Ser9-phosphorylated GSK-3β) were potential biomarkers for MCI in diabetic patients, with the accuracy as 0.76, 0.72, 0.66, 0.79, respectively. To improve the diagnostic accuracy, the combined four biomarkers showed the diagnostic accuracy of 83%. Diabetic patients with MCI presented more susceptibility to ApoE ε4 gene when compared with diabetic patients without MCI. Besides, six ApoE genotypes including ε2ε2, ε3ε3, ε4ε4, ε2ε3, ε2ε4, and ε3ε4 were detected in included patients, showing that the prevalence of ε3ε4 genotype was higher while ε3ε3 was lower in T2DM-MCI group. However, other ApoE genotypes presented no obvious difference in T2DM-MCI group and T2DM group. Thus, aging, activation of GSK-3β, ApoE ε4 expression and increased olfactory score are diagnostic for MCI in T2DM patients, and combination of these biomarkers can improve the diagnostic accuracy. However, inconsistent results were reported by Jacobson *et al.* [114]. They reported that ACE gene and ApoE ε4 gene were not related with increased risk of cognitive impairment in T1DM patients during 18-year follow up. Although current evidence is limited, consistent results were reported in different studies that the genetic factors influencing the risk of cognitive decline in patients with diabetes. Future studies should validate above findings in a larger amount of population, and the accuracy measurements of each parameter or biomarker are highly recommended.

**Table 6:** Details about the studies focusing on the relationship between genetic factors and cognitive function in diabetic patients

Study	Population	Design	Number	Mean age	Genetic factors	Cognitive measure	Association with cognition
Guerrero-Berroa et al [108]	T2DM; Israel	Cross-sectional, observational	793	72.8 ± 4.5	Haptoglobin 1-1	MMSE; CDR	HP 1-1 carriers associated with higher risk of cognitive decline
Wang et al et al [24]	T2DM; China	Cross-sectional, observational	167	60.15 ± 7.47	RAGE Gly82Ser	MoCA; DST; TMT; CDR; CDT; ST; VFT	No association
Tian et al [111]	T2DM; China	Cross-sectional, observational	210	60.19 ± 0.6	I/D gene of ACE; ACE level and activity	MoCA; DST; VFT; CDT; ST; TMT; SCWT; AVLT;	Increased ACE level and activity in cognitive decline; No association for ACE I/D gene and cognition
Xu et al [113]	T2DM; China	Cross-sectional, observational	694	74.92 ± 6.44	ApoE ε2; ApoE ε3; ApoE ε4 allele	MMSE; CDR	diagnostic accuracy of ApoE ε4 gene for MCI: 0.72
Jacobson et al [114]	T1DM; US	Retrospective, observational	1093	45.7 ± 6.8	ApoE (rs7412; rs429358); ACE (rs4340)	WAIS; DVT; FAS; TMT	No association

Notes: DST: digit symbol test; TMT: Trail Making Test-Part B; VFT: Verbal Fluency Test; MMSE: Mini-Mental State Examination; MCI: mild cognitive impairment; AVLT: Auditory Verbal Learning Test; CDR: Clinical Dementia Rating; SCWT: Stroop Color Words Test; FAS: Verbal Fluency Test; WAIS: Wechsler Adult Intelligence Scale; DVT: Digit Vigilance Test; CDT: the clock drawing test; ST: similarities test.

## CONCLUSIONS

In conclusion, we summarized available evidences on risk factors for cognitive decline in diabetic patients. The biomarkers or risk factors of cognitive decline in diabetic patients could be classified into the following three aspects: serum molecules or relevant complications, functional or metabolic changes by neuroimaging tools, and genetic variants. Specifically, factors related to poor glucose metabolism, insulin resistance, inflammation, comorbid depression, micro-/macrovascular complications, adipokines, neurotrophic molecules and Tau protein presented significant changes in diabetic patients with cognitive decline. Neuroimaging platform could provide more clues on the structural, functional and metabolic changes during the cognitive decline progression of diabetic patients. Genetic factors related to cognitive decline showed inconsistency based on the limited studies. Future studies should validate above findings in a larger population in order to find valuable, sensitive and specific biomarkers on early diagnosis for cognitive decline in diabetic patients.
